# Supplementary material for: An exported protein-interacting complex involved in the trafficking of virulence determinants in Plasmodium-infected erythrocytes
Source: Nat Commun. 2017 Jul 10;8:16044. doi: 10.1038/ncomms16044 (PMC5508133; doi:10.1038/ncomms16044)
Supplement: Supplementary Information [file ncomms16044-s1.pdf]

File Name: Supplementary Information
Description: Supplementary Figures and Supplementary Tables

File Name: Peer Review File
Description:

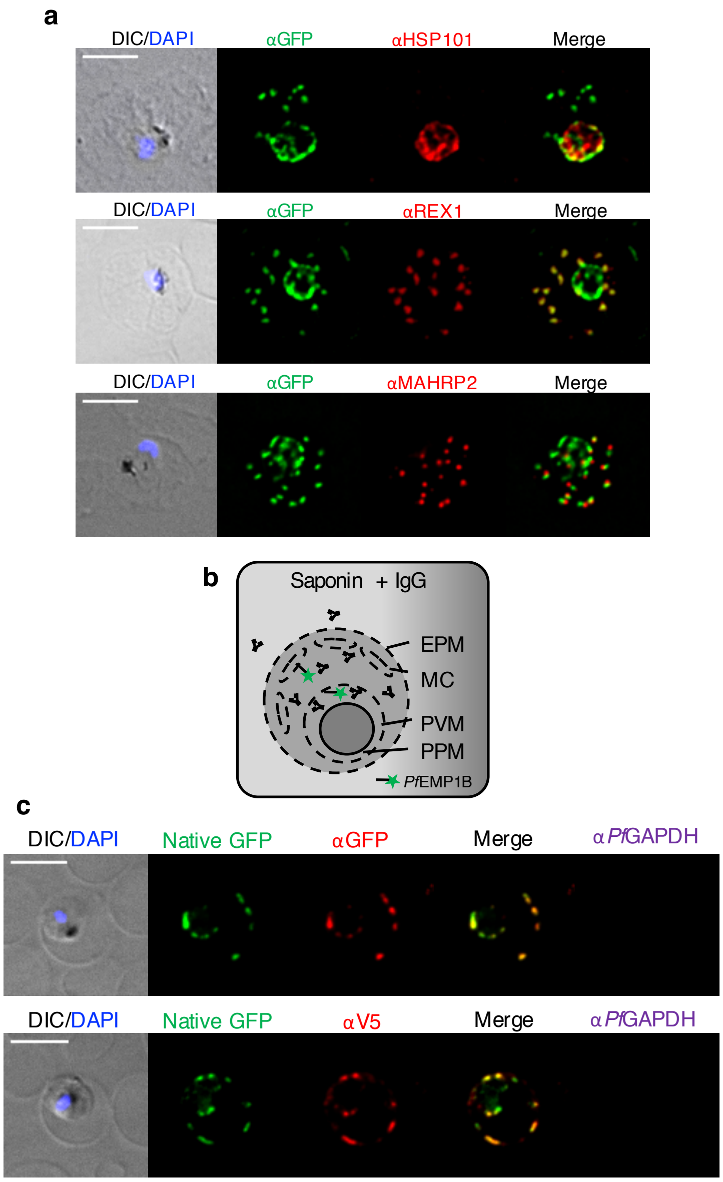

Supplementary Figure 1 | Immunofluorescence and proximity ligation microscopy reveal *Pf*EMP1B organisation

(a) Immunofluorescence analysis of *Pf*EMP1B-infected RBCs labelled with antisera recognising GFP (green) and HSP101 or REX1 or MAHRP2 (red). **(b)** Schematic of antibody access in saponin-permeabilised *Pf*EMP1B-infected RBCs. Cells were treated with saponin and lightly fixed before antibody (IgG) labelling. EPM, erythrocyte plasma membrane; MC, Maurer's clefts; PVM, parasitophorous vacuole membrane; PPM, parasite plasma membrane. **(c)** Immunofluorescence analysis of saponin-permeabilised *Pf*EMP1B-infected RBCs labelled with antisera recognising GFP or V5 (red). Native GFP fluorescence is shown in green. *Pf*GAPDH is shown as a control, indicating that the PPM is not breached. **(a, c)** DIC images and parasite nuclei (stained with DAPI; blue) are shown on the left. Scale bars = 5 μm.

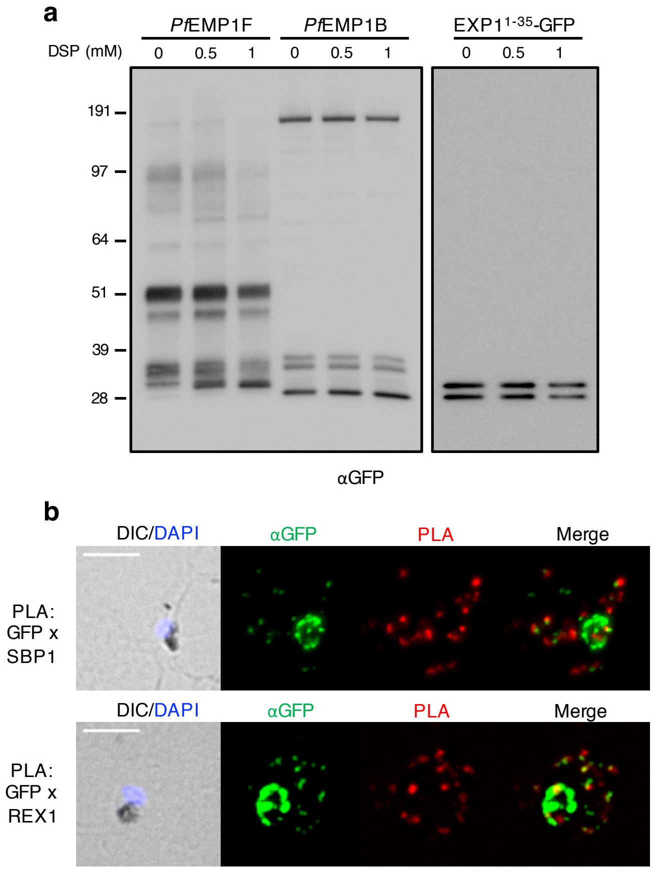

Supplementary Figure 2 | Immunoprecipitation of *Pf*EMP1B and interactions with Maurer's cleft proteins

(a) Immunoprecipitation (IP) using GFP-Trap® of *Pf*EMP1F, *Pf*EMP1B and EXP1$^{1\text{-}35}$-GFP (see Fig. 2b) parasite lysates after crosslinking with increasing concentrations of DSP. Eluates were prepared for Western blot and probed with anti-GFP. Molecular masses are shown in kDa. **(b)** Proximity ligation analysis (PLA) of *Pf*EMP1B-infected RBCs labelled with antisera recognising GFP and SBP1 or REX1. PLA signal is only produced if primary epitopes are ≤40 nm apart. DIC and DAPI (blue), anti-GFP (green) and PLA (red) are shown. Scale bars = 5 μm.

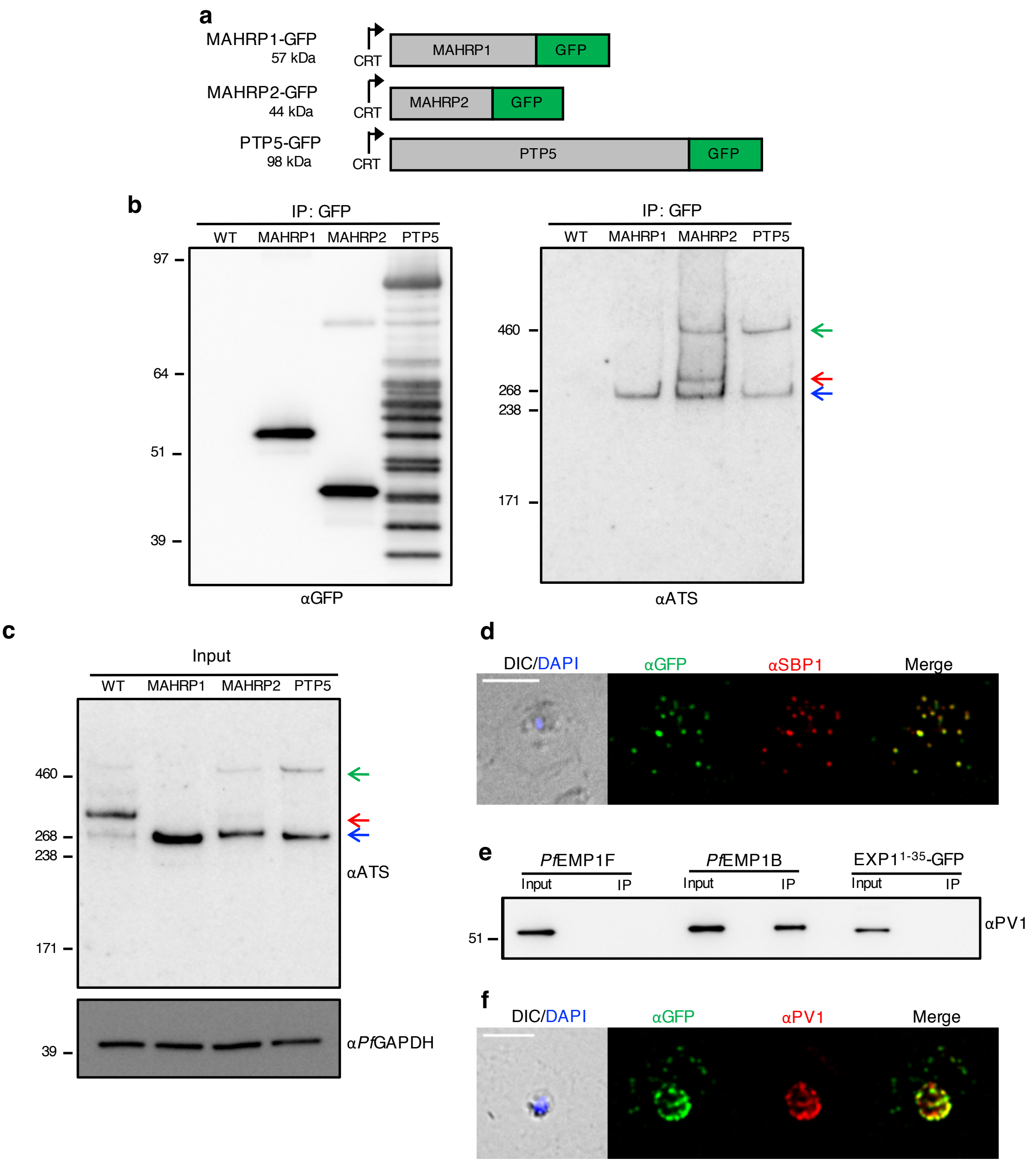

Supplementary Figure 3 | Confirmation of *Pf*EMP1 interactions identified in mass spectrometric analysis of *Pf*EMP1B IP

(a) Schematic of exported GFP-fusion proteins used in IP experiments. MAHRP1, MAHRP2 and PTP5 were fused to GFP and expressed in infected RBCs under the control of the CRT promoter. Sizes of fusion proteins are indicated. **(b, c)** Immunoprecipitation of MAHRP1-GFP, MAHRP2-GFP and PTP5-GFP parasite lysates using GFP-Trap®. **(b)** Eluates were prepared for Western blot and probed with anti-GFP and anti-*Pf*EMP1 (ATS) antisera. **(c)** Total protein (input) was prepared for Western blot and probed with anti-*Pf*EMP1 (ATS) and anti-GAPDH antisera. **(b, c)** Arrows indicate different *Pf*EMP1 variants. **(d)** Immunofluorescence analysis of PTP5-GFP infected RBCs labelled with antisera recognising GFP (green) and SBP1 (red). **(e)** Immunoprecipitation of *Pf*EMP1F, *Pf*EMP1B and EXP1$^{1\text{-}35}$-GFP transfectants using GFP-Trap®. Total protein (input) and eluate (IP) were prepared for Western blot and probed with anti-PV1 antisera. **(f)** Immunofluorescence analysis of *Pf*EMP1B-infected RBCs labelled with antisera recognising GFP (green) and PV1 (red).

(b,c,e) Molecular masses are shown in kDa. **(d,f)** DIC and DAPI (blue) are shown on the left. Scale bars = 5 μm.

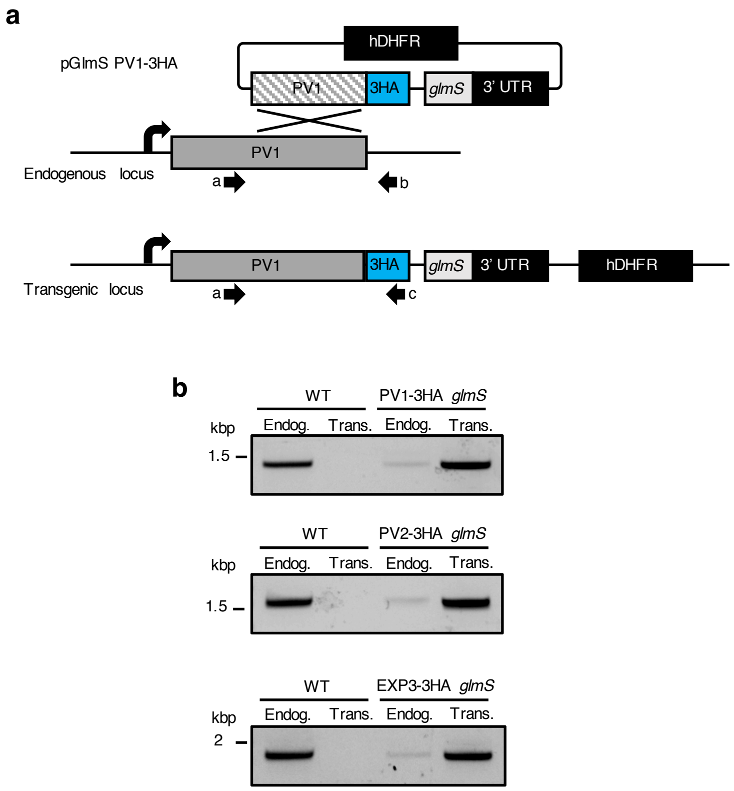

Supplementary Figure 4 | Generation of PV1-HA, PV2-HA and EXP3-HA *glmS* knockdown parasite transfectants

(a) Schematic of targeting construct designed to integrate into the endogenous *Pfpv1* locus by single crossover recombination. Arrows indicate positions of oligonucleotides used to confirm integration. hDHFR, human dihydrofolate reductase; 3HA, 3xHA epitope tag; *glmS*; *glmS* ribozyme. Similar constructs were generated to integrate into the *Pfpv2* and *Pfexp3* loci. **(b)** Diagnostic PCR analysis of wildtype (WT) and PV1, PV2 and EXP3 knockdown transfectant parasite lines using oligonucleotides targeting sites a, b and c. Oligonucleotide pair a/b amplifies the endogenous locus (Endog.) whilst oligonucleotide pair a/c amplifies the transgenic locus (Trans.). DNA size is shown in kbp.

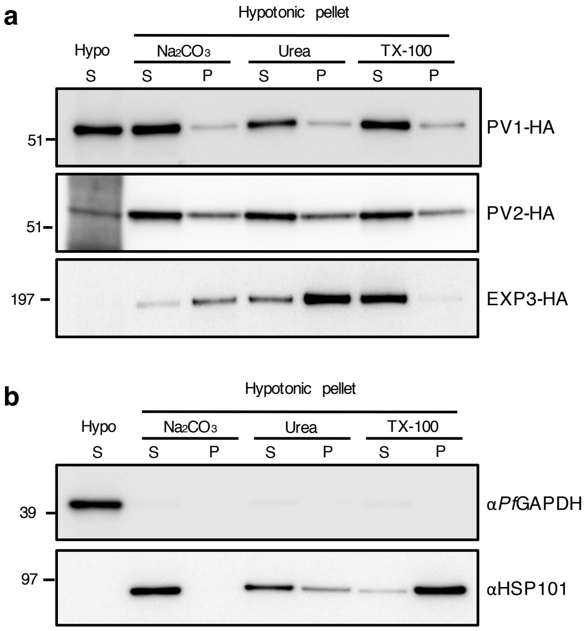

Supplementary Figure 5 | Solubility analysis of PV1-HA, PV2-HA and EXP3-HA
(a,b) Solubility analysis of purified PV1-HA, PV2-HA and EXP3-HA infected RBCs. After hypotonic release of soluble proteins (Hypo), protein pellets were subjected to treatment with sodium carbonate (Na_2CO_3), urea or TX-100. **(a)** Western blots of resulting supernatants (S) and pellets (P) were probed with anti-HA or, **(b)** anti-GAPDH and anti-HSP101 antisera. **(a,b)** Molecular masses are shown in kDa.

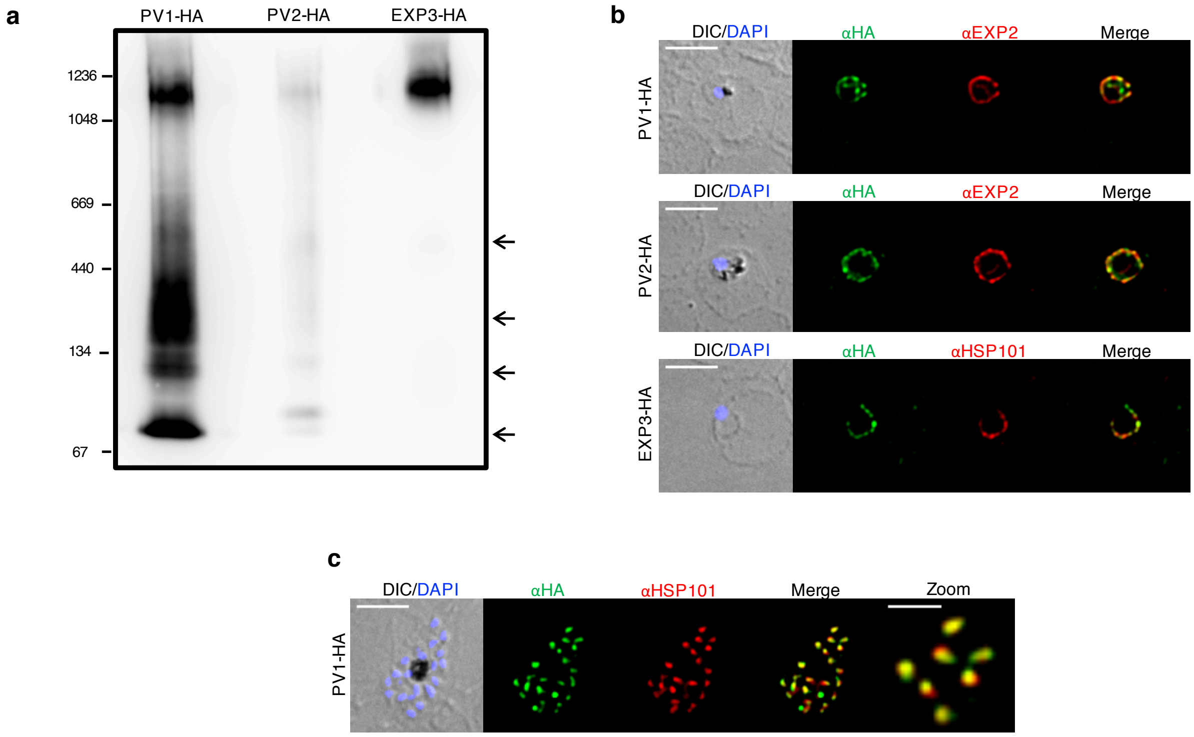

Supplementary Figure 6 | EPIC and PTEX co-localise in the PV and in the dense granules

(a) Full-length blot showing smaller protein complexes from blue native (BN)-PAGE analysis of PV1-HA, PV2-HA and EXP3-HA infected RBCs in Fig 5a. Western blots were probed with anti-HA antisera. Arrows indicate different size protein complexes. Molecular masses are shown in kDa. **(b, c)** Immunofluorescence analysis of RBCs infected with PV1-HA, PV2-HA and EXP3-HA transfectants at the early trophozoite **(b)** and schizont **(c)** stages, labelled with antisera recognising HA (green) and HSP101 or EXP2 (red). **(b,c)** DIC and DAPI (blue) are shown on the left. Scale bars = 5 μm. Zoom scale bar = 1 μm

a

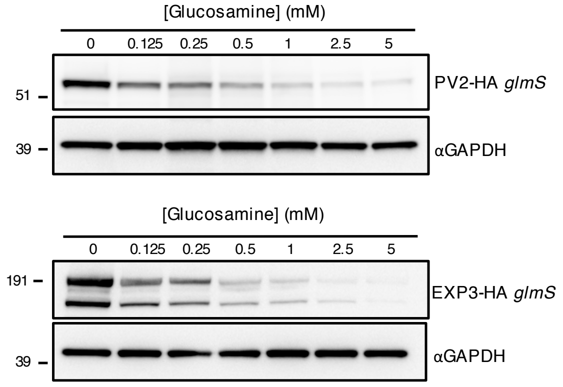

b

	[Glucosamine] (mM)						
**PV1-HA *glmS***	**0**	**0.125**	**0.25**	**0.5**	**1**	**2.5**	**5**
**αHA**	100	69	42	32	21	12	6
**αGAPDH**	100	102	94	99	95	86	77

	[Glucosamine] (mM)						
**PV2-HA *glmS***	**0**	**0.125**	**0.25**	**0.5**	**1**	**2.5**	**5**
**αHA**	100	46	35	20	9	5	3
**αGAPDH**	100	122	137	123	100	83	74

	[Glucosamine] (mM)						
**EXP3-HA *glmS***	**0**	**0.125**	**0.25**	**0.5**	**1**	**2.5**	**5**
**αHA**	100	45	36	18	10	4	2
**αGAPDH**	100	106	66	71	108	95	87

Supplementary Figure 7 | Quantitation of glucosamine-mediated knockdown of PV1-HA, PV2-HA and EXP3-HA

(a) PV2-HA and EXP3-HA *glmS* parasite-infected RBCs treated with increasing concentrations of glucosamine (GlcN) from the trophozoite stage of the previous cycle. Western blots (anti-HA) confirm knockdown of expression of PV2-HA and EXP3-HA. Molecular masses are shown in kDa. **(b)** Densitometry of anti-HA and anti-GAPDH signals in PV1-HA, PV2-HA and EXP3-HA knockdown Westerns shown in Figure 7a and Supplementary Figure 7a. Signal intensities are represented as a percentage of the anti-HA and anti-GAPDH signal intensities in untreated samples (0 mM GlcN).

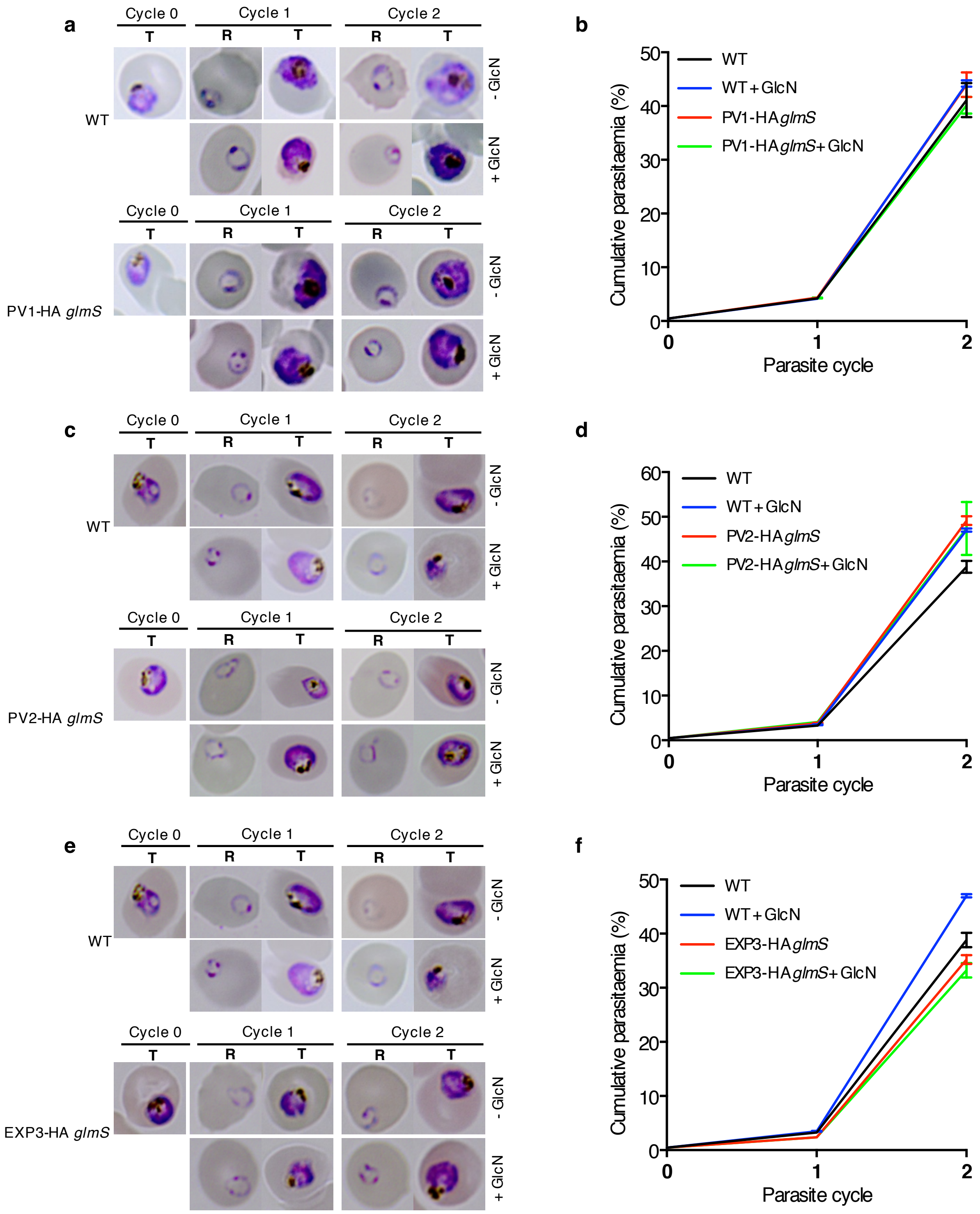

Supplementary Figure 8 | Knockdown of PV1-HA, PV2-HA and EXP3-HA does not affect parasite viability *in vitro*

Analysis of PV1-HA, PV2-HA and EXP3-HA parasite growth after knockdown. Parasite cultures were synchronised to a 2-hour window and treated with 0 mM or 2.5 mM GlcN at the mid-trophozoite-stage. **(a,c,e)** Giemsa smears of representative infected RBCs every 24 hours following addition of GlcN at 30-32 hours post-invasion (h.p.i.) (Cycle 0) in wildtype (WT) or PV1-HA *glmS*, PV2-HA *glmS* and EXP3-HA *glmS* parasite lines. R = ring-stage, T= trophozoite-stage. **(b,d,f)** Parasitaemia of treated cultures was determined every 48 hours

(starting from Cycle 1 trophozoite-stage) using flow cytometry (3 technical triplicates). Cumulative % parasitaemia ± S.E.M. was determined by taking into account parasite sub-culturing during the assay.

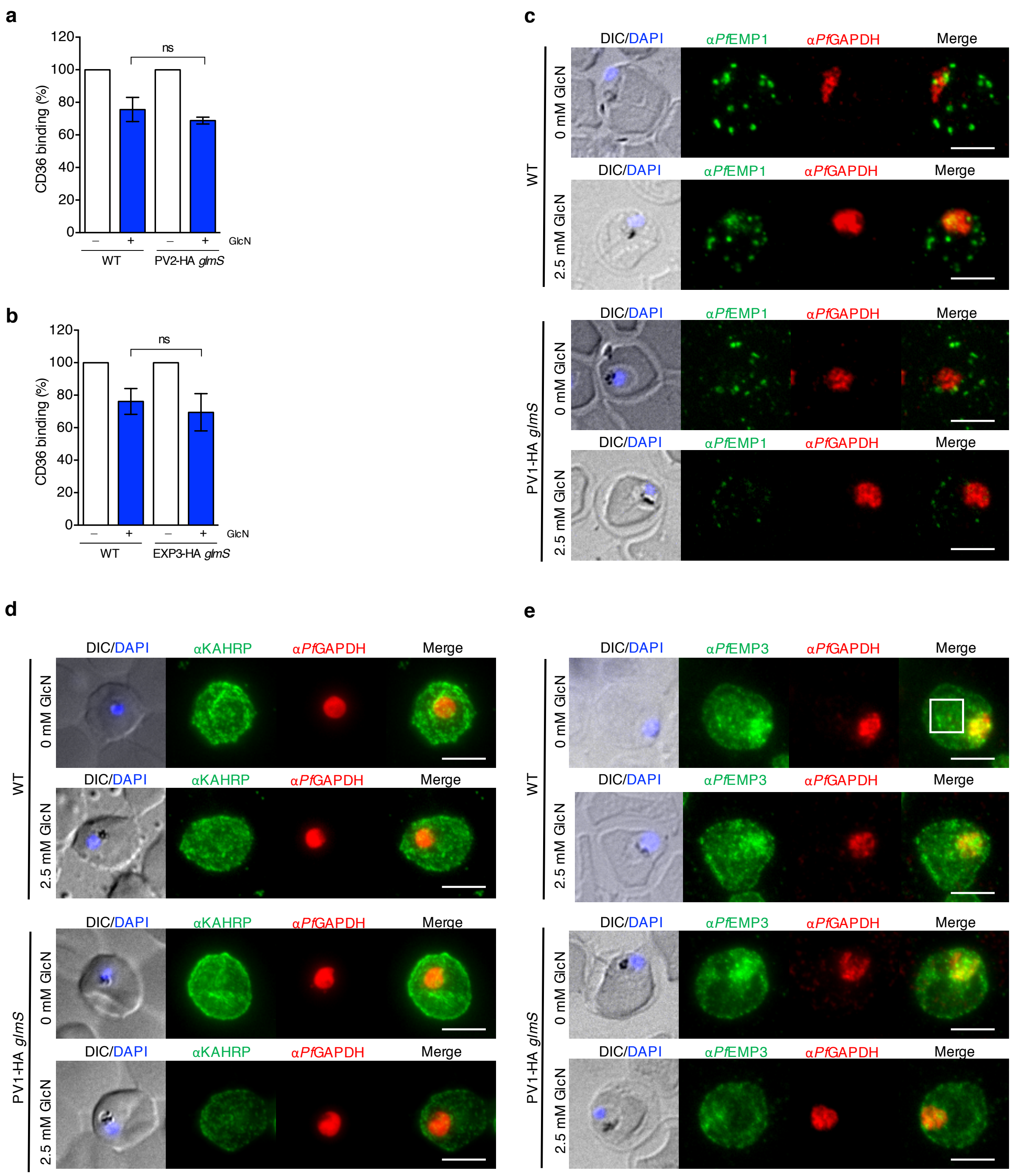

Supplementary Figure 9 | Effects of EPIC protein knockdown on protein export and cell properties in PV1-HA, PV2-HA and EXP3-HA *glmS* infected RBCs
(a,b) Adherence of wildtype (WT; A4), PV2-HA and EXP3-HA *glmS* infected RBCs (30-32 h.p.i.) to recombinant CD36 under flow conditions (0.1 Pa) measured in 10 pre-determined fields (binding indicates % of non-treated control + S.E.M.). GlcN treatment of the A4 line causes a modest decrease in adhesion, but there is no significant difference between the GlcN-treated parent and the transfectants (PV2 n = 3, p = 0.425, unpaired t-test; EXP3 n =3, p = 0.659, unpaired t-test). **(c-e)** Immunofluorescence analysis of WT (3D7) and PV1-HA *glmS*-infected RBCs at 23-26 h.p.i. following treatment with or without 2.5 mM GlcN. Parasites were labelled with **(c)** anti-*Pf*EMP1, **(d)** anti-KAHRP and **(e)** anti-*Pf*EMP3 (green) and anti-*Pf*GAPDH (red) antisera. DIC and DAPI (blue) are shown on the left. Fluorescence

quantitation was performed on the whole cell for *Pf*EMP1 and KAHRP and in a region in the host cytoplasm for *Pf*EMP3 (as indicated by the box). Scale bars = 5 μm.

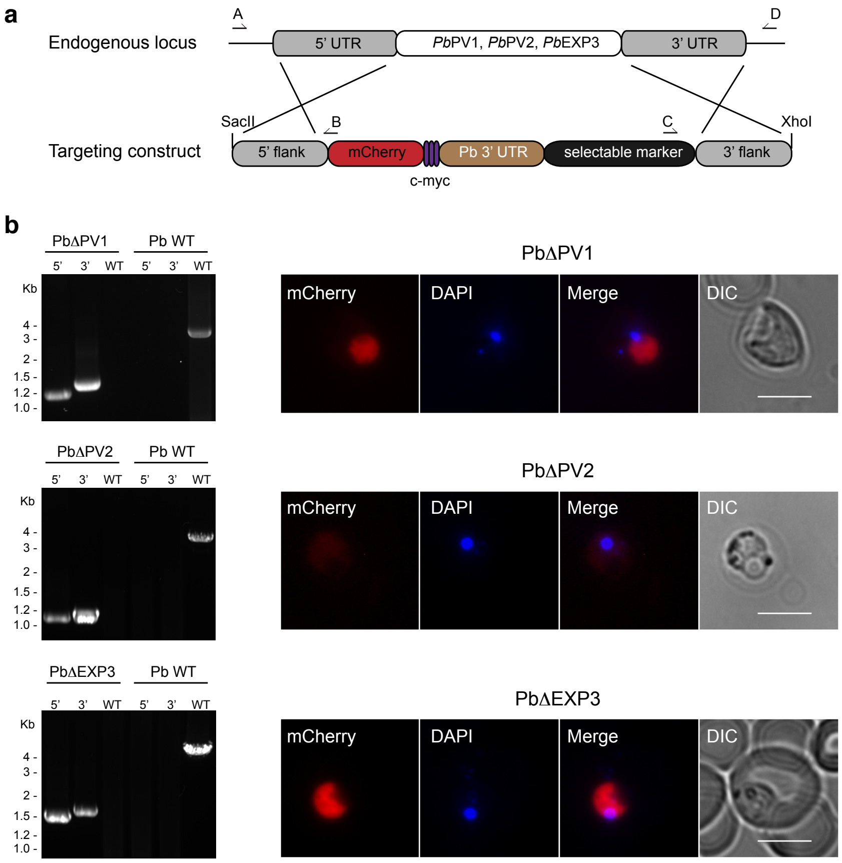

Supplementary Figure 10 | Generation of *P. berghei* EPIC component knockout parasites

(a) Schematic of targeting construct used to generate *Pb*ΔPV1, *Pb*ΔPV2 and *Pb*ΔEXP3. Integration results in replacement of the gene with the vector backbone containing mCherry, 3Myc (3x c-Myc tag), a non-endogenous 3'UTR, and *Tg*DHFR-TS (*Toxoplasma gondii* dihydrofolate reductase-thymidylate synthase) as the selectable marker. **(b)** Diagnostic PCR analysis of *Pb*ANKA (wildtype) and *Pb*ΔPV1, *Pb*ΔPV2 and *Pb*ΔEXP3 parasites using oligonucleotides targeting sites A, B, C and D. Oligonucleotide pair A/B amplifies the 5' transgenic locus (5'), oligonucleotide pair C/D amplifies the 3' transgenic locus (3') whilst oligonucleotide pair A/D amplifies the endogenous locus (WT). DNA size is shown in kbp. **(c)** Live cell imaging of *Pb*ΔPV1, *Pb*ΔPV2 and *Pb*ΔEXP3 infected RBCs showing mCherry (red) fluorescence. DAPI (blue) and DIC are shown. Scale bars = 5 µm.

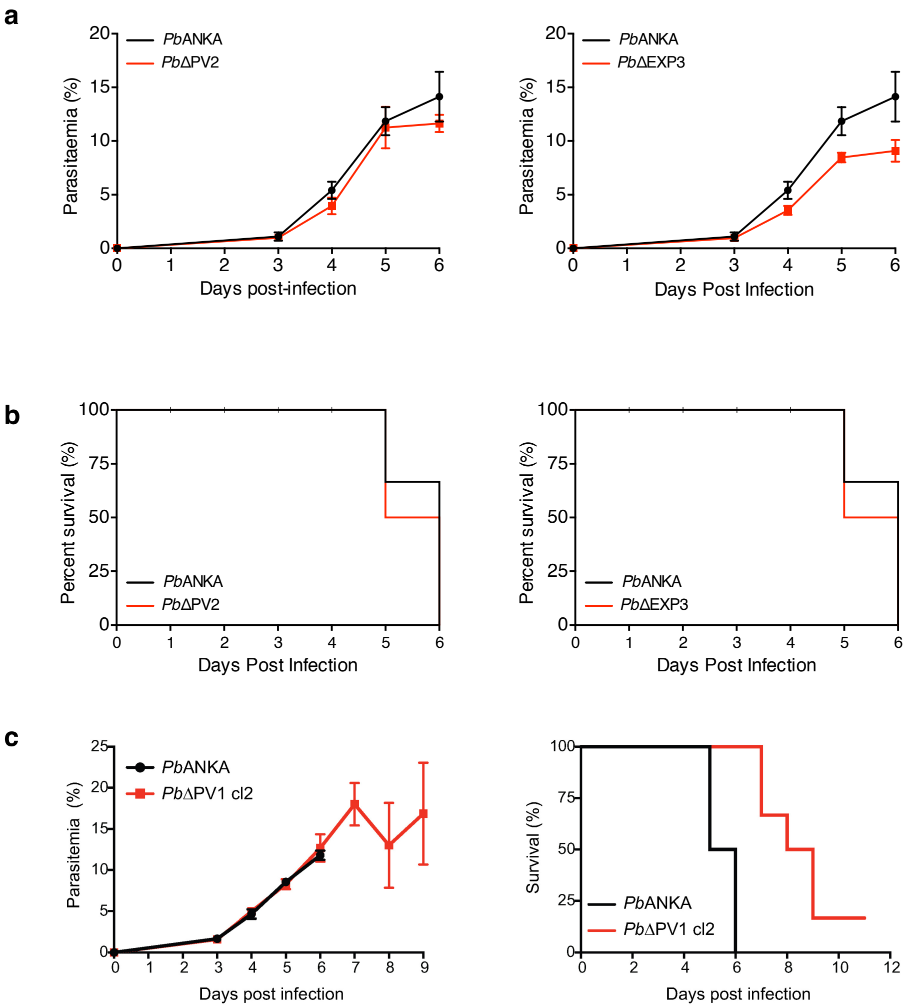

Supplementary Figure 11 | Genetic disruption of PV1 does, but PV2 and EXP3 does not, affect virulence of *P. berghei* infections

(a) Parasitaemia and **(b)** survival curves of C57/BL6 mice (n = 6 *Pb*ANKA, *Pb*ΔPV2, *Pb*ΔEXP3) after intraperitoneal administration of $1x10^6$ *Pb*ANKA, *Pb*ΔPV2 or *Pb*ΔEXP3 parasites. **(a)** No significant difference in parasitaemia after infection with *Pb*ANKA and *Pb*ΔPV2 or *Pb*ΔEXP3 parasites was observed using an unpaired *t*-test **(b)** Survival of *Pb*ΔPV2 and *Pb*ΔEXP3 infected mice was similar to wildtype *Pb*ANKA infected mice as determined by log-rank test. Death indicates mice succumbing to cerebral malaria. **(c)** Second experiment showing survival of *Pb*ΔPV1 infected mice (n=6) was increased in comparison with *Pb*ANKA infected mice (n=6; $p < 0.0001$, log-rank (Mantel-Cox) test). Death indicates mice succumbing to cerebral malaria.

Supplementary Figure 12 | Full length Western blots

Figure 1d

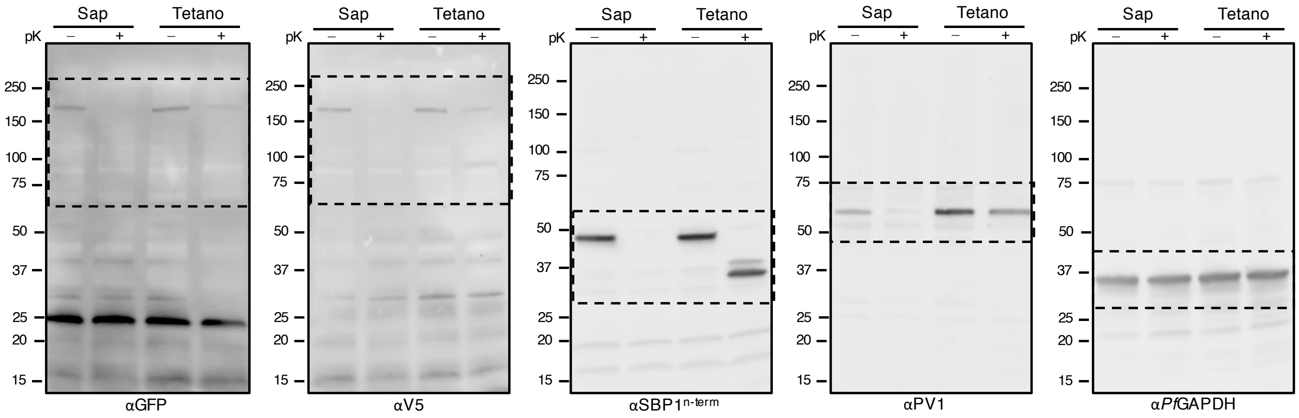

Figure 2c

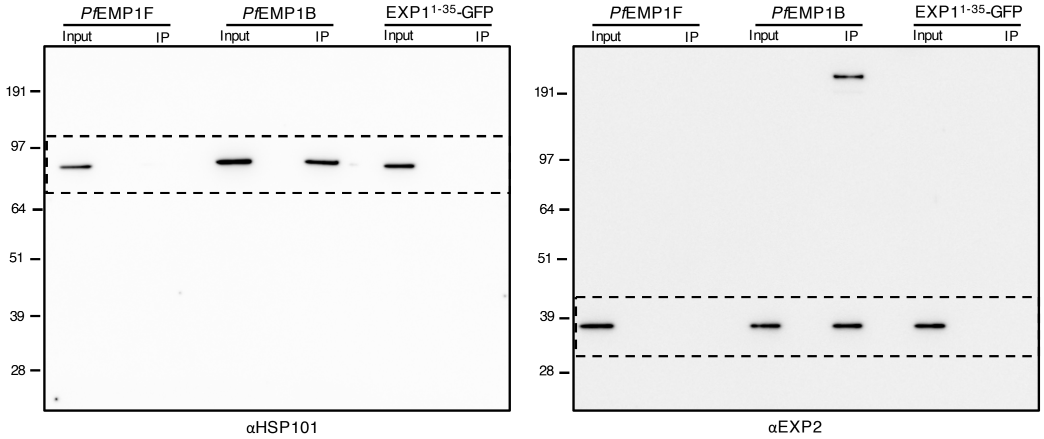

Figure 2d

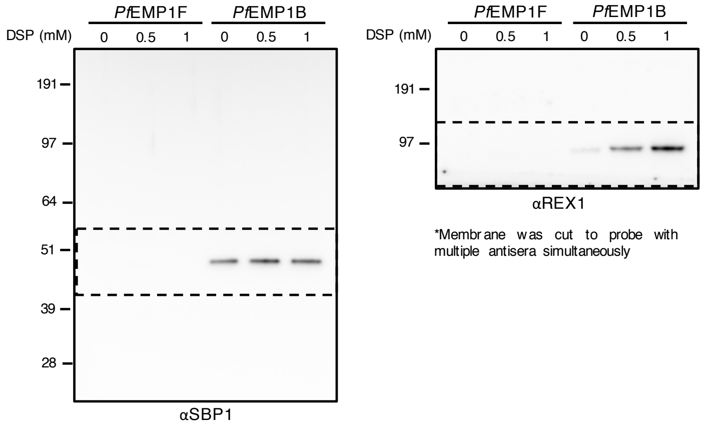

Figure 2e

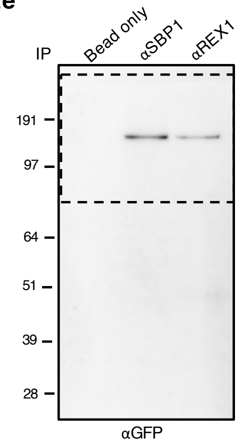

Figure 5a

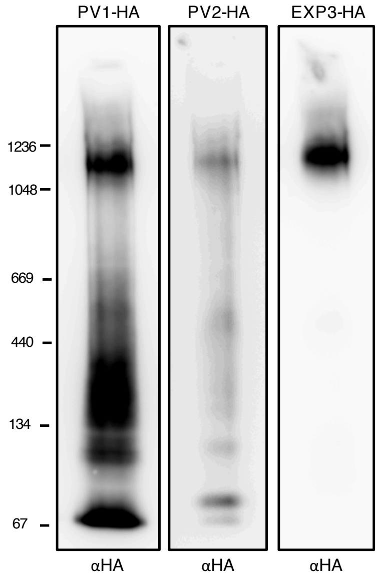

Figure 5b

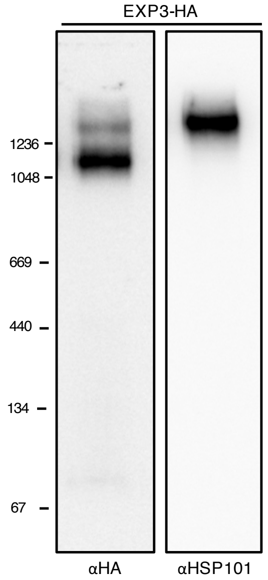

Figure 5d

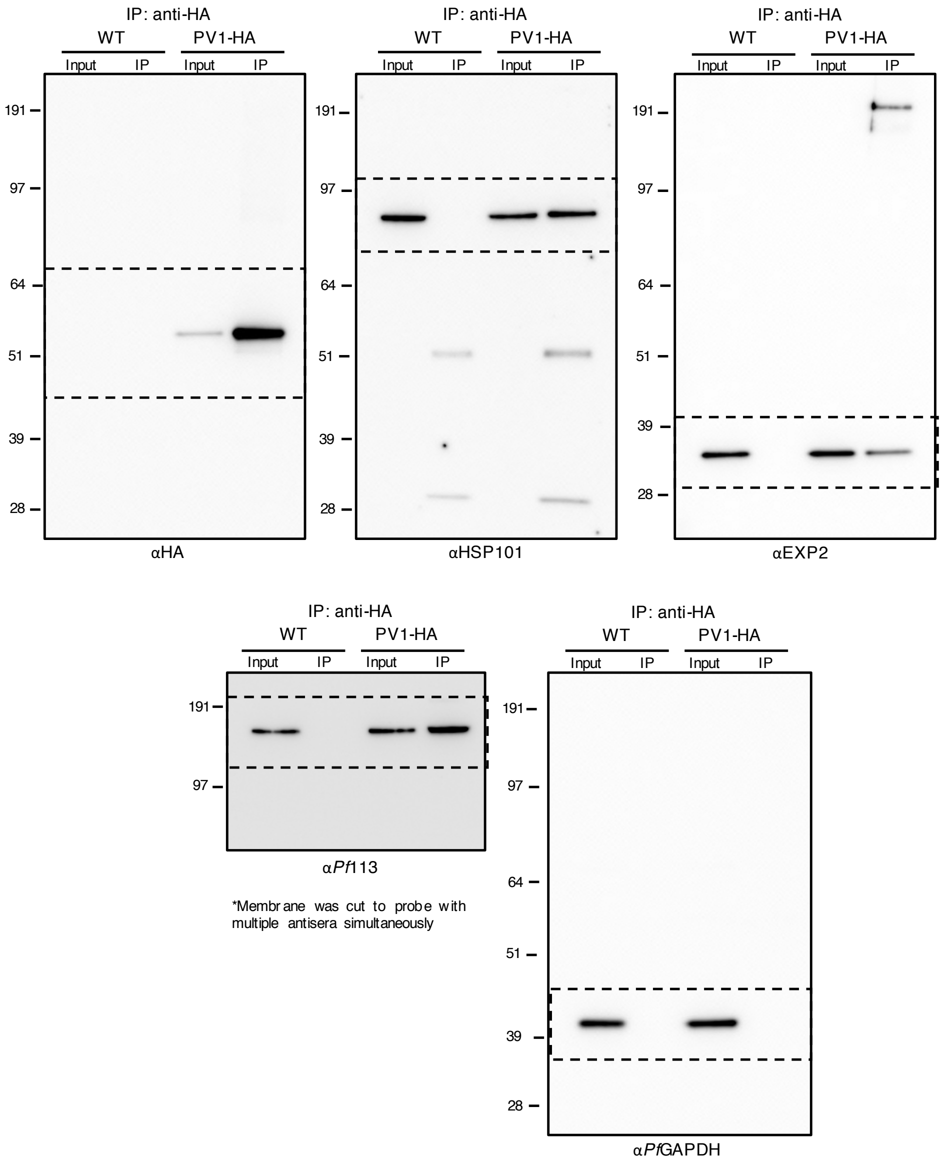

Figure 5e

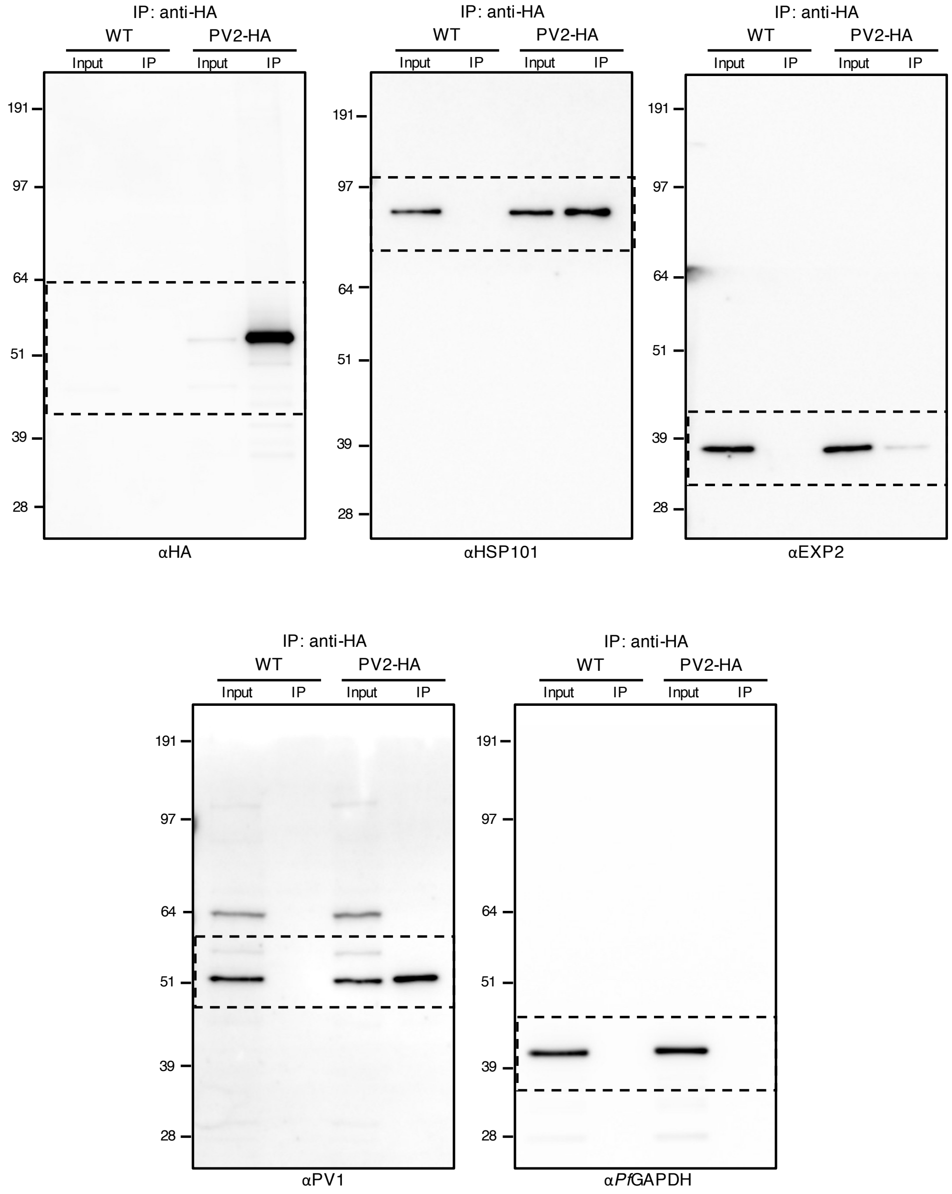

Figure 5f

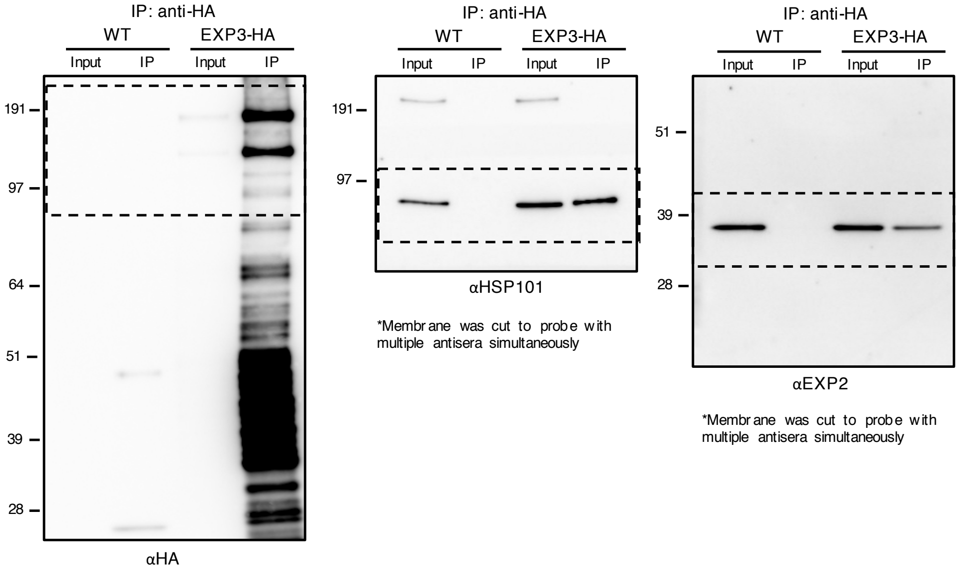

Figure 5f contd.

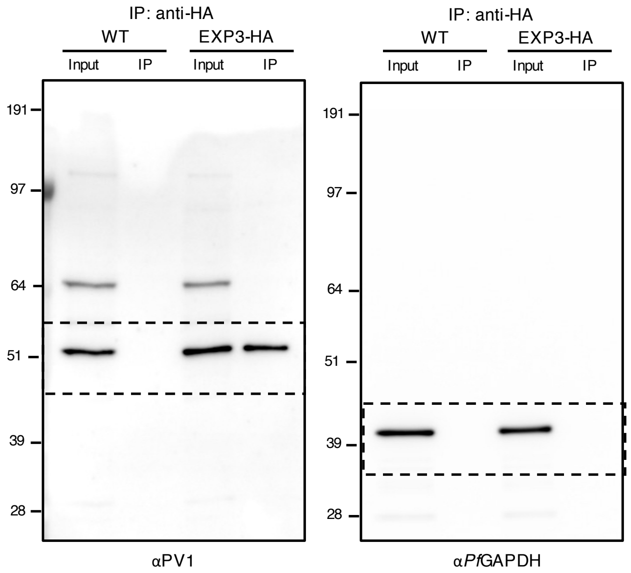

Figure 6e

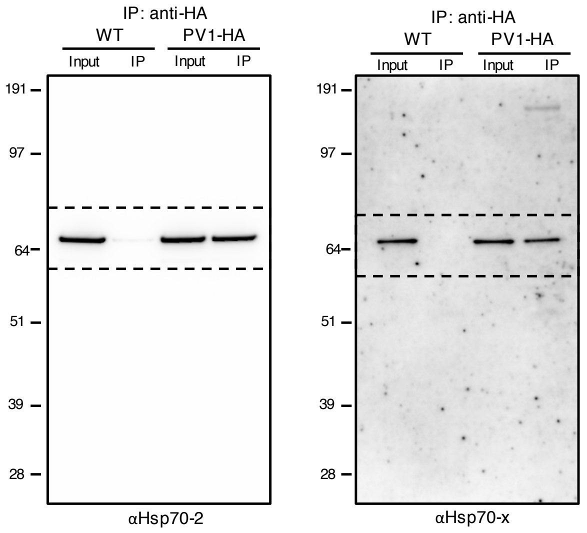

Figure 7a

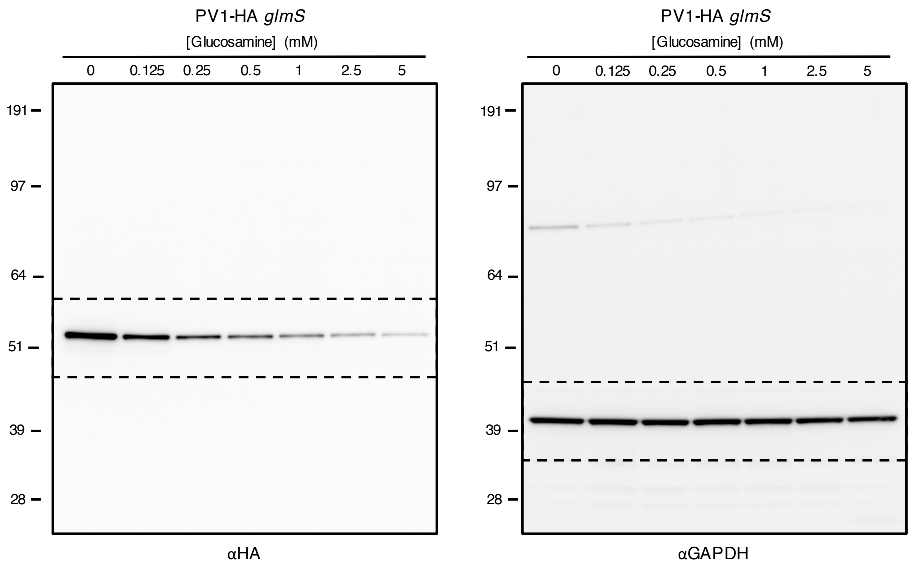

Gene ID	Annotation	Number of significant ms/ms spectra								
		Experiment 1				Experiment 2				Avg. fold change
		*Pf*EMP1B		*Pf*EMP1F		*Pf*EMP1B		*Pf*EMP1F		
		**1**	**2**	**1**	**2**	**1**	**2**	**1**	**2**	
-	*Pf*EMP1B	203	192	0	0	179	210	0	0	**>196**
-	*Pf*EMP1F	0	0	123	188	0	0	99	83	**>123.25**
PF3D7_1024800	conserved Plasmodium protein, unknown function	18	22	0	0	29	36	0	0	**>26.25**
PF3D7_1116800	heat shock protein 101 (HSP101)	6	4	0	0	28	34	1	1	**>18**
PF3D7_1222300	endoplasmin, putative (GRP94)	10	10	0	0	18	17	0	0	**>13.75**
PF3D7_1436300	translocon component PTEX150 (PTEX150)	8	6	0	0	14	19	0	0	**>11.75**
PF3D7_1129100	parasitophorous vacuolar protein 1 (PV1)	29	29	3	5	38	44	4	2	**10**
PF3D7_0629200	DnaJ protein, putative	2	4	1	1	12	15	1	0	**>8.25**
PF3D7_0721100	conserved Plasmodium protein, unknown function	8	4	0	0	9	9	0	0	**>7.5**
PF3D7_1318800	secretory complex protein 63 (SEC63)	17	12	2	2	13	17	2	2	**7.375**
PF3D7_1344200	heat shock protein 110, putative (HSP110)	3	2	0	0	8	10	0	0	**>5.75**
PF3D7_1439800	vesicle-associated membrane protein, putative	9	8	2	2	8	8	2	0	**5.5**
PF3D7_1105600	translocon component PTEX88 (PTEX88)	3	2	0	0	7	9	0	0	**>5.25**
PF3D7_1010700	dolichyl-phosphate-mannose protein mannosyltransferase, putative	5	5	0	0	4	4	0	0	**>4.5**
PF3D7_0936300	ring-exported protein 3 (REX3)	4	2	0	0	3	7	0	0	**>4**
PF3D7_1016400	serine/threonine protein kinase, FIKK family (FIKK 10.1)	3	1	0	0	4	6	0	0	**>3.5**
PF3D7_1438100	secretory complex protein 62 (SEC62)	3	6	0	0	3	2	0	0	**>3.5**
PF3D7_1404900	conserved Plasmodium protein, unknown function	2	1	0	0	4	5	0	0	**>3**
PF3D7_1108700	heat shock protein DnaJ homologue, Pfj2	0	0	0	0	3	6	0	0	**>2.25**
PF3D7_0501200	parasite-infected erythrocyte surface protein 2 (PIESP2)	1	1	0	0	3	3	0	0	**>2**
PF3D7_1226900	conserved Plasmodium protein, unknown function	1	1	0	0	3	3	0	0	**>2**
PF3D7_1135400	conserved Plasmodium protein, unknown function	1	0	0	0	2	5	0	0	**>2**
PF3D7_1311800	M1-family alanyl aminopeptidase (M1AAP)	1	1	0	0	2	4	0	0	**>2**
PF3D7_0501300	skeleton-binding protein 1 (SBP1)	2	1	0	0	3	2	0	0	**>2**
PF3D7_0716300	conserved Plasmodium protein, unknown function	1	4	0	0	2	1	0	0	**>2**
PF3D7_0112800	Pfmc-2TM Maurer's cleft two transmembrane protein	2	1	0	0	3	1	0	0	**>1.75**
PF3D7_0935900	ring-exported protein 1 (REX1)	0	0	0	0	3	4	0	0	**>1.75**
PF3D7_1471100	exported protein 2 (EXP2)	0	0	0	0	2	3	0	0	**>1.25**
PF3D7_0925900	conserved Plasmodium protein, unknown function	0	0	0	0	3	2	0	0	**>1.25**
PF3D7_0928600	conserved Plasmodium protein, unknown function	0	0	0	0	2	3	0	0	**>1.25**
PF3D7_1345100	thioredoxin 2 (TRX2)	1	0	0	0	2	2	0	0	**>1.25**
PF3D7_1370300	membrane associated histidine-rich protein 1 (MAHRP1)	1	1	0	0	1	2	0	0	**>1.25**
PF3D7_1108600	endoplasmic reticulum-resident calcium binding protein (ERC)	1	1	0	0	2	1	0	0	**>1.25**
PF3D7_1320000	rhoptry protein 2, putative (PRP2)	0	1	0	0	1	2	0	0	**>1**
PF3D7_1002100	EMP1-trafficking protein 5 (PTP5)	0	0	0	0	3	1	0	0	**>1**
PF3D7_0831400	Plasmodium exported protein, unknown function	0	0	0	0	2	2	0	0	**>1**
PF3D7_1353200	membrane associated histidine-rich protein 2 (MAHRP2)	0	0	0	0	2	2	0	0	**>1**
PF3D7_1134100	protein disulfide isomerase (PDI-11)	0	0	0	0	2	1	0	0	**>0.75**
PF3D7_1239700	cell division protein FtsH, putative	0	0	0	0	1	2	0	0	**>0.75**
PF3D7_1033200	early transcribed membrane protein 10.2 (ETRAMP 10.2)	0	0	0	0	0	2	0	0	**>0.5**
	**False discovery rate (FDR)**	**0.46%**	**0.26%**	**0.11%**	**1.06%**	**0.91%**	**0.75%**	**0.83%**	**0.88%**	

Supplementary Table 1 | Mass spectrometry analysis of *Pf*EMP1B parasite interacting proteins

Parasite proteins that are unique to, or ≥ 5-fold enriched in, *Pf*EMP1B co-IP compared to control *Pf*EMP1F. PlasmoDB gene ID, protein name and number of peptides detected in two independent experiments are shown.

Gene ID	Annotation	Number of significant ms/ms spectra								
		Experiment 1				Experiment 2				Avg. fold change
		*Pf*EMP1B		*Pf*EMP1F		*Pf*EMP1B		*Pf*EMP1F		
		1	2	1	2	1	2	1	2	
TCPA_HUMAN	T-complex protein 1 subunit alpha	3	3	0	0	14	15	0	0	**>8.75**
TCPG_HUMAN	T-complex protein 1 subunit gamma	5	8	0	0	12	10	0	0	**>8.75**
TCPQ_HUMAN	T-complex protein 1 subunit theta	4	4	0	0	14	12	0	0	**>8.5**
TCPB_HUMAN	T-complex protein 1 subunit beta	1	0	0	0	13	13	0	0	**>6.75**
TCPZ_HUMAN	T-complex protein 1 subunit zeta	1	1	0	0	12	12	0	0	**>6.5**
TCPD_HUMAN	T-complex protein 1 subunit delta	3	2	0	0	9	7	0	0	**>5.25**
TCPH_HUMAN	T-complex protein 1 subunit eta	1	2	0	0	7	9	0	0	**>4.75**
TCPE_HUMAN	T-complex protein 1 subunit episilon	1	3	0	0	7	7	0	0	**>4.5**
HS90A_HUMAN	Heat shock protein HSP90 alpha	0	2	0	0	5	5	0	0	**>3**
PRS6A_HUMAN	26S protease regulatory subunit 6A	0	1	0	0	4	6	0	0	**>2.75**
PRS10_HUMAN	26S protease regulatory subunit 10B	1	1	0	0	5	4	0	0	**>2.75**
PRS7_HUMAN	26S protease regulatory subunit 7	1	1	0	0	4	4	0	0	**>2.5**
PSMD2_HUMAN	26S proteasome non-ATPase regulatory subunit 2	0	0	0	0	4	6	0	0	**>2.5**
CAH2_HUMAN	Carbonic anhydrase 2	0	0	0	0	3	3	0	0	**>1.5**
PSDE_HUMAN	26S proteasome non-ATPase regulatory subunit 14	1	0	0	0	3	2	0	0	**>1.5**
KT3K_HUMAN	Ketosamine-3-kinase	1	1	0	0	2	2	0	0	**>1.5**
PRS4_HUMAN	26S protrase regulatory subunit 4	0	0	0	0	4	1	0	0	**>1.25**
PSD12_HUMAN	26S proteasome non-ATPase regulatory subunit 12	0	0	0	0	2	3	0	0	**>1.25**
DDI2_HUMAN	Protein DDI1 homolog 2	1	0	0	0	1	3	0	0	**>1.25**
PRS6B_HUMAN	26S protease regulatory subunit 6B	0	0	0	0	3	2	0	0	**>1.25**
PSD11_HUMAN	26S proteasome non-ATPase regulatory subunit 11	0	0	0	0	2	2	0	0	**>1**
PSMD3_HUMAN	26S proteasome non-ATPase regulatory subunit 3	0	0	0	0	2	2	0	0	**>1**
DNPEP_HUMAN	Aspartyl aminopeptidase	0	0	0	0	2	2	0	0	**>1**
H3BTE6_HUMAN	Erythrocyte membrane protein band 4.2	0	0	0	0	1	2	0	0	**>0.75**
K2C8_HUMAN	Keratin, type II cytoskeletal 8	0	0	0	0	2	1	0	0	**>0.75**
RPIA_HUMAN	Ribose-5-phosphate isomerase	0	0	0	0	2	0	0	0	**>0.5**
	**False discovery rate (FDR)**	**0.46%**	**0.26%**	**0.11%**	**1.06%**	**0.91%**	**0.75%**	**0.83%**	**0.88%**	

Supplementary Table 2 | Mass spectrometry analysis of *Pf*EMP1B human interacting proteins

Human proteins that are unique to, or ≥ 5-fold enriched in, *Pf*EMP1B co-IP compared to control *Pf*EMP1F. UniProt gene ID, protein name and number of peptides detected in two independent experiments are shown.

Gene ID	Annotation	Number of significant ms/ms spectra								
		Experiment 1				Experiment 2				Avg. fold change
		PV1-HA		WT		PV1-HA		WT		
		1	2	1	2	1	2	1	2	
PF3D7_1129100	parasitophorous vacuolar protein 1 (PV1)	97	120	0	0	94	105	0	0	**>104**
PF3D7_1024800	exported protein 3 (EXP3)	90	107	0	0	68	77	0	0	**>85.5**
PF3D7_0917900	heat shock protein 70 (HSP70-2)	60	64	0	0	84	83	0	0	**>72.75**
PF3D7_1222300	endoplasmin, putative (GRP94)	24	27	0	0	33	34	0	0	**>29.5**
PF3D7_1436300	translocon component PTEX150 (PTEX150)	36	32	0	0	22	26	0	0	**>29**
PF3D7_0831700	heat shock protein 70 (HSP70-x)	27	31	0	0	16	17	0	0	**>22.75**
PF3D7_1116800	heat shock protein 101 (HSP101)	20	27	0	0	22	21	0	0	**>22.5**
PF3D7_1420700	surface protein *Pf*113 (*Pf*113)	21	18	0	0	12	18	0	0	**>17.25**
PF3D7_0827900	protein disulfide isomerase (PDI8)	7	4	0	0	25	24	0	0	**>15**
PF3D7_1226900	parasitophorous vacuolar protein 2 (PV2)	13	15	0	0	10	12	0	0	**>12.5**
PF3D7_1464600	phosphatase, putative	11	9	0	0	13	15	0	0	**>12**
PF3D7_1228600	merozoite surface protein 9 (MSP9)	13	14	0	0	8	5	0	0	**>10**
PF3D7_1454400	aminopeptidase P (APP)	9	13	1	1	7	7	1	1	**9**
PF3D7_0930300	merozoite surface protein 1 (MSP1)	12	12	0	0	2	3	0	0	**>7.25**
PF3D7_1105600	translocon component PTEX88 (PTEX88)	10	11	0	0	2	2	0	0	**>6.25**
PF3D7_0902800	serine repeat antigen 9 (SERA9)	9	12	0	0	0	0	0	0	**>5.25**
PF3D7_1033200	early transcribed membrane protein 10.2 (ETRAMP 10.2)	4	7	0	0	3	6	0	0	**>5**
PF3D7_0721100	conserved Plasmodium protein, unknown function	8	7	0	0	3	2	0	0	**>5**
PF3D7_0929400	high molecular weight rhoptry protein 2 (RhopH2)	2	2	0	0	11	5	0	0	**>5**
PF3D7_1016300	glycophorin binding protein (GBP)	5	9	0	0	3	2	0	0	**>4.75**
PF3D7_0207600	serine repeat antigen 5 (SERA5)	7	8	0	0	2	2	0	0	**>4.75**
PF3D7_1311800	M1-family alanyl aminopeptidase (M1AAP)	8	5	0	0	2	4	0	0	**>4.75**
PF3D7_0104200	StAR-related lipid transfer protein	6	6	0	0	4	2	0	0	**>4.5**
PF3D7_1471100	exported protein 2 (EXP2)	4	7	0	0	3	4	0	0	**>4.5**
PF3D7_1116700	cathepsin C, homolog, dipeptidyl aminopeptidase 1 (DPAP1)	7	6	0	0	4	1	0	0	**>4.5**
PF3D7_0730900	EMP1-trafficking protein (PTP4)	2	2	0	0	4	7	0	0	**>3.75**
PF3D7_0406200	sexual stage-specific protein precursor (Pfs16)	3	5	0	0	2	2	0	0	**>3**
PF3D7_0716300	conserved Plasmodium protein, unknown function	4	4	0	0	2	1	0	0	**>2.75**
PF3D7_0501200	parasite-infected erythrocyte surface protein 2 (PIESP2)	3	4	0	0	2	1	0	0	**>2.5**
PF3D7_1016400	serine/threonine protein kinase, FIKK family (FIKK 10.1)	2	4	0	0	2	2	0	0	**>2.5**
PF3D7_0112200	multidrug resistance-associated protein 1 (MRP1)	5	5	0	0	0	0	0	0	**>2.5**
PF3D7_1104400	conserved Plasmodium protein, unknown function	2	2	0	0	4	2	0	0	**>2.5**
PF3D7_0215000	acyl-CoA synthetase (ACS9)	4	5	0	0	0	0	0	0	**>2.25**
PF3D7_0532100	early transcribed membrane protein 5 (ETRAMP5)	3	3	0	0	2	1	0	0	**>2.25**
PF3D7_0532400	lysine-rich membrane-associated PHISTb protein (LyMP)	3	3	0	0	1	2	0	0	**>2.25**
PF3D7_1462300	conserved Plasmodium protein, unknown function	2	1	0	0	3	3	0	0	**>2.25**
PF3D7_1135400	conserved Plasmodium protein, unknown function	2	3	0	0	2	1	0	0	**>2**
PF3D7_1334500	MSP7-like protein (MSRP6)	3	4	0	0	0	0	0	0	**>1.75**
PF3D7_0207800	serine repeat antigen 3 (SERA3)	3	4	0	0	0	0	0	0	**>1.75**
PF3D7_0905400	high molecular weight rhoptry protein 3 (RhopH3)	2	3	0	0	1	1	0	0	**>1.75**
PF3D7_1001500	early transcribed membrane protein 10.1 (ETRAMP10.1)	1	1	0	0	2	3	0	0	**>1.75**
PF3D7_1252700	Plasmodium exported protein (PHISTb), unknown function	3	2	0	0	1	1	0	0	**>1.75**
PF3D7_1408000	plasmepsin II	3	3	0	0	0	0	0	0	**>1.5**
PF3D7_0829200	prohibitin, putative	3	3	0	0	0	0	0	0	**>1.5**

PF3D7_0925900	conserved Plasmodium protein, unknown function	2	2	0	0	0	1	0	0	**>1.25**
PF3D7_1419200	thioredoxin-like protein, putative	2	1	0	0	1	1	0	0	**>1.25**
PF3D7_0936000	ring-exported protein 2 (REX2)	1	2	0	0	1	1	0	0	**>1.25**
PF3D7_0830400	conserved Plasmodium protein, unknown function	1	2	0	0	1	1	0	0	**>1.25**
PF3D7_1121600	exported protein 1 (EXP1)	3	0	0	0	1	1	0	0	**>1.25**
PF3D7_0811600	conserved Plasmodium protein, unknown function	2	2	0	0	0	0	0	0	**>1**
PF3D7_0912400	alkaline phosphatase, putative	2	2	0	0	0	0	0	0	**>1**
PF3D7_0500800	mature parasite-infected erythrocyte surface antigen (MESA)	2	2	0	0	0	0	0	0	**>1**
PF3D7_1335100	merozoite surface protein 7 (MSP7)	2	1	0	0	0	0	0	0	**>0.75**
PF3D7_0104500	conserved Plamsodium protein, unknown function	1	2	0	0	0	0	0	0	**>0.75**
PF3D7_0104400	4-hydroxy-3-methylbut-2-enyl diphosphate reductase (LytB)	2	1	0	0	0	0	0	0	**>0.75**
PF3D7_1364100	6-csyteine protein (P92)	1	2	0	0	0	0	0	0	**>0.75**
PF3D7_0913700	conserved Plasmodium protein, unknown function	1	2	0	0	0	0	0	0	**>0.75**
	**False discovery rate (FDR)**	**0.31%**	**0.63%**	**1.48%**	**0.25%**	**0.67%**	**0.90%**	**0.00%**	**0.38%**	

Supplementary Table 3 | Mass spectrometry analysis of PV1-HA interacting proteins
Proteins that are unique to, or ≥ 5-fold enriched in, PV1-HA co-IP compared to control wildtype (WT). PlasmoDB gene ID, protein name and number of peptides detected in two independent experiments are shown.

Gene ID	Annotation	Number of significant ms/ms spectra								
		Experiment 1				Experiment 2				Avg. fold change
		PV2		WT		PV2		WT		
		1	2	1	2	1	2	1	2	
PF3D7_1226900	parasitophorous vacuolar protein 2 (PV2)	34	20	0	0	60	53	0	0	**>41.75**
PF3D7_1129100	parasitophorous vacuolar protein 1 (PV1)	17	14	1	1	24	23	1	1	**19.50**
PF3D7_1024800	exported protein 3 (EXP3)	3	0	0	0	24	21	0	0	**>12.00**
PF3D7_1116800	heat shock protein 101 (HSP101)	0	0	0	0	8	5	0	0	**>3.25**
PF3D7_1201000	Plasmodium exported protein (PHISTb), unknown function	2	1	0	0	5	4	0	0	**>3.00**
PF3D7_1228600	merozoite surface protein 9 (MSP9)	0	0	0	0	4	3	0	0	**>1.75**
PF3D7_1436300	translocon component PTEX150 (PTEX150)	0	0	0	0	3	4	0	0	**>1.75**
PF3D7_0501200	parasite-infected erythrocyte surface protein 2 (PIESP2)		0	0	0	3	2	0	0	**>1.67**
PF3D7_1105600	translocon component PTEX88 (PTEX88)	0	0	0	0	3	2	0	0	**>1.25**
PF3D7_1016400	serine/threonine protein kinase, FIKK family (FIKK 10.1)	0	0	0	0	2	2	0	0	**>1.00**
PF3D7_1471100	exported protein 2 (EXP2)	0	0	0	0	1	2	0	0	**>0.75**
	**False discovery rate (FDR)**	**0.38%**	**0.25%**	**0.43%**	**0.49%**	**0.92%**	**0.26%**	**0.70%**	**0.00%**	

Supplementary Table 4 | Mass spectrometry analysis of PV2-HA interacting proteins
Proteins that are unique to, or ≥ 5-fold enriched in, PV2-HA co-IP compared to control wildtype (WT). PlasmoDB gene ID, protein name and number of peptides detected in two independent experiments are shown

Gene ID	Annotation	Number of significant ms/ms spectra								
		Experiment 1				Experiment 2				Avg. fold change
		EXP3		WT		EXP3		WT		
		1	2	1	2	1	2	1	2	
PF3D7_1024800	exported protein 3 (EXP3)	75	60	0	0	84	118	0	0	**>84.25**
PF3D7_1129100	parasitophorous vacuolar protein 1 (PV1)	27	19	1	1	45	72	1	1	**>40.75**
PF3D7_1436300	translocon component PTEX150 (PTEX150)	3	1	0	0	12	20	0	0	**>9**
PF3D7_1226900	parasitophorous vacuolar protein 2 (PV2)	1	1	0	0	12	14	0	0	**>7**
PF3D7_1116700	cathepsin C, homolog, dipeptidyl aminopeptidase 1 (DPAP1)	3	3	1	0	6	10	1	0	**>5.5**
PF3D7_1116800	heat shock protein 101 (HSP101)	2	2	0	0	7	10	0	0	**>5.25**
PF3D7_1454400	aminopeptidase P (APP)	0	0	0	0	7	11	0	0	**>4.5**
PF3D7_0902800	serine repeat antigen 9 (SERA9)	0	0	0	0	6	6	0	0	**>3**
PF3D7_1016300	glycophorin binding protein (GBP)	0	0	0	0	7	5	0	0	**>3**
PF3D7_1016400	serine/threonine protein kinase, FIKK family (FIKK 10.1)	1	1	0	0	4	5	0	0	**>2.75**
PF3D7_1105600	translocon component PTEX88 (PTEX88)	1	0	0	0	3	6	0	0	**>2.5**
PF3D7_1201000	Plasmodium exported protein (PHISTb), unknown function	1	2	0	0	3	4	0	0	**>2.5**
PF3D7_0104200	StAR-related lipid transfer protein	0	0	0	0	4	5	0	0	**>2.25**
PF3D7_0305300	conserved Plasmodium membrane protein, unknown function	2	0	0	0	2	3	0	0	**>1.75**
PF3D7_0501200	parasite-infected erythrocyte surface protein 2 (PIESP2)	0	0	0	0	3	3	0	0	**>1.5**
PF3D7_0925900	conserved Plasmodium protein, unknown function	0	0	0	0	3	3	0	0	**>1.5**
PF3D7_1222300	endoplasmin, putative (GRP94)	0	0	0	0	3	3	0	0	**>1.5**
PF3D7_1302300	Plasmodium exported protein, unknown function	0	0	0	0	3	2	0	0	**>1.25**
PF3D7_0532100	early transcribed membrane protein 5 (ETRAMP5)	0	0	0	0	2	3	0	0	**>1.25**
PF3D7_1320000	rhoptry protein 2, putative (PRP2)	2	0	0	0	2	1	0	0	**>1.25**
PF3D7_1321900	conserved Plasmodium protein, unknown function	1	0	0	0	1	3	0	0	**>1.25**
PF3D7_1471100	exported protein 2 (EXP2)	0	0	0	0	2	3	0	0	**>1.25**
PF3D7_1228600	merozoite surface protein 9 (MSP9)	0	0	0	0	2	2	0	0	**>1**
PF3D7_1420700	surface protein *Pf*113 (*Pf*113)	0	0	0	0	1	3	0	0	**>1**
PF3D7_1451800	sortillin, putative	0	0	0	0	2	2	0	0	**>1**
PF3D7_0827900	protein disulfide isomerase (PDI8)	0	0	0	0	2	2	0	0	**>1**
PF3D7_1404900	conserved Plasmodium protein, unknown function	0	0	0	0	2	2	0	0	**>1**
PF3D7_0721100	conserved Plasmodium protein, unknown function	0	0	0	0	1	2	0	0	**>0.75**
PF3D7_0406200	sexual stage-specific protein precursor (Pfs16)	0	0	0	0	0	3	0	0	**>0.75**
PF3D7_0830400	conserved Plasmodium protein, unknown function	0	0	0	0	1	2	0	0	**>0.75**
PF3D7_1334500	MSP7-like protein (MSRP6)	0	0	0	0	1	2	0	0	**>0.75**
PF3D7_0911900	falstatin (ICP)	0	0	0	0	2	1	0	0	**>0.75**
PF3D7_1135400	conserved Plasmodium protein, unknown function	0	0	0	0	1	2	0	0	**>0.75**
PF3D7_1464600	phosphatase, putative	0	0	0	0	2	1	0	0	**>0.75**
PF3D7_0215000	acyl-CoA synthetase (ACS9)	0	0	0	0	1	2	0	0	**>0.75**
PF3D7_0207600	serine repeat antigen 5 (SERA5)	0	0	0	0	1	2	0	0	**>0.75**
PF3D7_1033200	early transcribed membrane protein 10.2 (ETRAMP 10.2)	0	0	0	0	0	2	0	0	**>0.5**
	**False discovery rate (FDR)**	**0.31%**	**0.63%**	**1.48%**	**0.25%**	**0.67%**	**0.90%**	**0.00%**	**0.38%**	

Supplementary Table 5 | Mass spectrometry analysis of EXP3-HA interacting proteins
Proteins that are unique to, or ≥ 5-fold enriched in, EXP3-HA co-IP compared to control wildtype (WT). PlasmoDB gene ID, protein name and number of peptides detected in two independent experiments are shown.

Primer name	Sequence
glmS PV1 forward	**GTCGAC**CCATCTGAAAATGATTTTTC
glmS PV1 reverse	**CTGCAG**GCTCGATATTGGTGTG
glmS PV2 forward	**GTCGAC**GAATTTATATCAGCTCC
glmS PV2 reverse	**CTGCAG**ATTTTCCGTAATATAATTTTG
glmS EXP3 forward	**GTCGAC**GAAGAAGAAAAAGAAGACG
glmS EXP3 reverse	**CTGCAG**AGAACTTAACCATGGAGC
PV1 5' int. screen	ATGATTAAAATAATATTAGCTAGC
PV2 5' int. screen	ATGTTTATAATAAAATGCATAGTTTTCG
EXP3 5' int. screen	CGAAACATCTAAGATGTGAAG
CRT MAHRP1 forward	**CTCGAG**ATGGCAGAGCAAGCAG
CRT MAHRP1 reverse	**GGTACC**ATTATCTTTTTTTTCTTGTTCTAA
CRT PTP5 forward	**CTCGAG**ATGGAAAACATAATAAACAAG
CRT PTP5 reverse	**GGTACC**TTTTAATTTCTTTTGAGATCTAC
*Pb*ΔPV1 5' UTR forward	GAT**CCGCGG**GAGGCTACCAATAAATCATAATG
*Pb*ΔPV1 5' UTR reverse	CAT**GTTAAC**TGCTAACGCCACCTTAATCAT
*Pb*ΔPV1 3' UTR forward	GAG**CCTAGG**TGTATTTTCGCGTCTGTACATTAG
*Pb*ΔPV1 3' UTR reverse	CAC**CTCGAG**GCACTACTAAATCTAGTAGATATG
*Pb*ΔPV2 5' UTR forward	GAT**CCGCGG**CCTTAGTCTTAAAACTTATGGC
*Pb*ΔPV2 5' UTR reverse	CAT**GTTAAC**AGCCATTTTGAAAAATAGTGTGAA
*Pb*ΔPV2 3' UTR forward	GAG**CCTAGG**AGTATATACACGCGCATGCAC
*Pb*ΔPV2 3' UTR reverse	CAC**CTCGAG**GGACAGCAACAAAGGATATAC
*Pb*ΔEXP3 5' UTR forward	GAT**CCGCGG**CCTGTGCGCAGTTTTAACACA
*Pb*ΔEXP3 5' UTR reverse	CAT**GTTAAC**GAGTTTTTTCTTCAATATCATTTTATCAG
*Pb*ΔEXP3 3' UTR forward	GAG**CCTAGG**ACATAAAGGAACTTCATTACCAG
*Pb*ΔEXP3 3' UTR reverse	CAC**CTCGAG**GAGCTAGCCAACAATTTACTC
*Pb*ΔPV1 'A' int. screen	CGTTTCCGGTGCATCATTTATG
*Pb*ΔPV1 'D' int. screen	TGTAACACATATAACTTGTTCAGG
*Pb*ΔPV2 'A' int. screen	TTAAGGCTGCTTTCTTTAATGTCCAT
*Pb*ΔPV2 'D' int. screen	CAAGTTGGCAACAAGGTAAATTG
*Pb*ΔEXP3 'A' int. screen	GAAAGGATACATACAATAAGGGATT
*Pb*ΔEXP3 'D' int. screen	GTAACAAAATGAGAGGAGGATTAC
Pb global 'B' int. screen	GCCATGTTATCCTCCTCGC
Pb global 'C' int. screen	CCGTGTGAATATGCTCATTTTG

Supplementary Table 6 | Oligonucleotides used in this study
Oligonucleotide name and sequence used in this study are shown. Bold indicates restriction endonuclease sites.
